# Emotional Reactions and Adaptation to COVID-19 Lockdown (or Confinement) by Spanish Competitive Athletes: Some Lesson for the Future

**DOI:** 10.3389/fpsyg.2021.621606

**Published:** 2021-05-26

**Authors:** José Carlos Jaenes Sánchez, David Alarcón Rubio, Manuel Trujillo, Rafael Peñaloza Gómez, Amir Hossien Mehrsafar, Andrea Chirico, Francesco Giancamilli, Fabio Lucidi

**Affiliations:** ^1^Department of Social Anthropology, Basic Psychology and Public Health, Universidad Pablo de Olavide, Seville, Spain; ^2^Andalusian Center of Sport Medicine (CAMD), Seville, Spain; ^3^School of Medicine, New York University, New York, NY, United States; ^4^Faculty of Higher Studies Zaragoza, National Autonomous University of Mexico, Mexico City, Mexico; ^5^Department of Sport Psychology, Faculty of Sport Sciences, University of Tehran, Tehran, Iran; ^6^Department of Social and Developmental Psychology, Faculty of Medicine and Psychology, University of Rome, “La Sapienza”, Rome, Italy

**Keywords:** COVID-19, confinement, training, stress, coping activities, mood/emotion, athletes, sport

## Abstract

The Coronavirus Covid 19 (SARS-CoV-2) pandemic has produced terrible effects in the world economy and is shaking social and political stability around the world. The world of sport has obviously been severely affected by the pandemic, as authorities progressively canceled all level of competitions, including the 2020 Olympic Games in Tokyo. In Spain, the initial government-lockdown closed the Sports High-performance Centers, and many other sports facilities. In order to support athlete's health and performance at crises like these, an online questionnaire named RECOVID-19, was designed to assess how athletes were living their lives during such periods of home confinement. The main purpose of the questionnaire was to assess the impact of prolongued confinement on athlete's psychological, emotional, and behavioral performance. One thousand, two hundred forty-eight athletes participated in the survey. They had the fllowing characteristics: (Mean age = 22.31 ± 11.49, Female: 53%), who compete at National (*N*: 1017, Mean age = 21.58 ± 11.42, Female: 52%) and International level (*N*: 231, Mean age = 25.56 ± 11.22, Female: 57%). Results showed that during the confinement period, those athletes who lacked motivation reported a higher level of stressful thoughts, more behavioral problems, and greater emotional upheaval (anger, fatigue, tension, and depression). However, those athletes who accepted confinement measures as necessary, and were in favor of respecting the rules of social isolation, fostered positive emotional states such as feelings of friendship. In addition, the availability of some sport equipment together with the ability to continue some training, were (1) protective factors against emotional stress, lack of motivation and behavioral problems; and (2) they were associated with greater respect for, and adherence to, confinement rules. Gender differences, tested by multigroup analysis, revealed that coping activities were more often associated to negative emotional states among women, whereas the ongoing availability of training information and future conditions were equally protective factors for both genders. This study also showed that receiving coaching, support and completing frequent training routines seem to be valuable tools to prevent or reduce some of the harmful effects of isolation on athlete's emotional well-being. The conclusions derived from this research would possibly help sport authorities to design supporting policies and plans to support athletes and trainers in future disruptive health crises.

## Introduction

As of March 20th, 2021, and according to the Faiser Family Foundation (www.kff.org consulted March 20, 2021), SARS-CoV-2Coronavirus (COVID-19) has infected 122.315647 million people and has taken 2,701.443 million lives all over the world. The pandemic is still expanding, though growing and effective vaccination programs are beginning to have some modulating effect on the pandemic morbidity and mortality as the proportion of vaccinated populations grow.

The pandemic is also severely affecting the world's economy, causing drastic reductions of Gross Domestic Product, massive loss of employment, and threatening social and political stability in many parts of the world. Spain, with over 3 million reported infections and more than 72 thousand deaths has also been severely affected by COVID-19 which it is causing an unprecedented economic slowdown (Zaar and Ávila, 2020). Drastic mobility restrictions, followed the initial lockdown (“confinement”), in an attempt to control the rapidly rising second wave of COVID-19, and its corollary of hospitals close to collapse, very high rates of infection, high morbidity and mortality among healthcare personnel, intense political polarization, and the growing social controversy and discontent of people who were demoralized by having to face additional mobility limitations, just after completing the prolonged (83 days) lockdown which followed the Spanish Government initial declaration of a State of Alarm on 14 March 2020 (Gobierno de España, 2020). The first 45 days of the state of alarm lockdown were the stricter, basically requiring all but essential workers, to stay at home and worked on-line. Schools, universities, and other educational centers were closed. The state of alarm limited free mobility of Spanish citizen, and it required them to stay home all day, except for essential activities such as food shopping or visiting health services, or working activities defined as crucial. Inevitably, the world of sport has also been severely affected by the pandemic (World Health Organization, 2020a,b).

Following international and national health's recommendations, both international and Spanish sports facilities were closed, including high performance and sports medical centers, specialized training centers, gyms, outdoor and indoor spectator sport activities, and even professional sport leagues. Athletes, trainers, coaches, and other support staff, as well as professionals (physicians, physiotherapists, psychologists, among others) were directed to stay at home as well. Local, regional, and national competitions in all sports were postponed early, and were canceled a short time later (Mann et al., 2020). Uncertainty was a common and popular word to describe the day-to-day situation, and the emotional climate. And not just among regular citizens, but even more in in the sports world, as key decisions were delayed generating further controversy. The International Olympic Committee (IOC) postponed any decision about the celebration of Olympic Games (OG), even when the Canadian Olympic (COC), and Paralympic Committee (CPC) announced that Canadian athletes would not attend the Tokyo 2020 Olympic Games (Donnelly, 2020). This was a move that generated considerable controversy between athletes, the IOC, and many sponsoring companies. Athletes argued for a voice in the decision making, driven by fear of contagion by exposure (Corsini et al., 2020). No one felt immune, despite the obvious general good fitness of such athletes as soccer players.

Finally, when the Olympics Games were postponed until 2021, the consequent emotional impact on the athletes, and their coaches may have been some relief from the confusion, but it surely lead to profound disappointment, frustration, and a broad spectrum of adverse emotional and cognitive reactions such as fear for their future careers as competitive athletes (Taku and Arai, 2020). Previous work on COVID-19 already indicated that athletes might face significant difficulties as a result of the severe training limitations, the adverse impact of canceled competitions and major events, and the loss of the strong motivation that accompanies the chance to compete in such unique events as the Tokyo Olympics Games (Mehrsafar et al., 2020). Compounding the problem, the sport economy was also deeply affected (García-Tascón et al., 2020). Making the future of the athletes financial and career support even more doubtful.

As the pandemic unfolded, we (Sports Psychologists in the Andalusian Center of Sport Medicine's Psychology Unit (ACSMPU) wanted to learn about the effects of the pandemic confinement on the lives of athletes of different specialty sports, and about potentially successful interventions that might modulate the adverse effects detected. The Spanish Rowing and Swimming Federation, and the Andalusian sports authorities, as well as sport clubs and independent coaches, contacted the Andalusian Center, seeking guidance on how to help and support their athletes during the pandemic' confinement. Given the scarcity of precedent guided advise, we found it necessary to get real-time information about the athlete's needs, concerns, and emotional reactions, during and after the period of confinement (World Health Organization, 2020c). The initial literature defined this period as a major stressful period possibly associated with emotional reactions typical of Post-Traumatic Stress Disorder (Sood, 2020); a position soon discarded, as reactions occurring during the confinement period could not, by definition, meet the key temporal criteria of Post-Traumatic Stress Disorder while occurring during the trauma (Jaenes et al., 2020b). So rather than pursuediagnostic entities, we opted to sample broad, cognitive, amotional, and behavioral parametrs with well-validated nstruments.

The Profile of Mood States (POMS) inventory, developed by McNair et al. (1971, 1992), has been widely used in sport and exercise research (Terry and Lane, 2000; Terry et al., 2003). Following Morgan's Mental Health Model, positive mood states enhance successful sports performance, whereas negative mood states is associated with a poorer performance (Morgan, 1980, 1985; Morgan et al., 1988). Numerous experimental studies and meta-analysis, have shown that athletes' negative mood states predict poorer performance in a wide range of sporting activities (Berger and Motl, 2000; Leunes and Burger, 2000; Lochbaum et al., 2021). However, the relationship between mood and physical activity can go in the opposite direction: the practice of sports activities improves mood, both in professional and amateur athletes (Steptoe and Bolton, 1988). In addition, the improvement of mood after physical activity is observed even in critical situations such as people suffering from a specific ailment or chronic disease (Ensari et al., 2017; Crush et al., 2018). For athletes, it has been observed that the practice of physical activity and sports during the recovery period of a physical injury reduces their negative moods and is thus associated with a more favorable recovery (Hadala et al., 2010).

Just as physical activity can improve mood, other coping activities might also reduce negative mood during a period of recovery or stress, such as leissure activities, social networking, artistic activities or learning activities (Waters and Moore, 2002; Drake et al., 2014; Domuschieva-Rogleva and Iancheva, 2017; Chan et al., 2019). It has been discussed whether the practice of these activities' directly influences mood states, or whether such influence is mediated via the reduction of stressful thoughts and the increase in motivation. A recent study in sport students shows that, among other coping strategies, positive cognitive restructuring of the COVID 19 pandemic situation was associated with positive mood states (Iancheva et al., 2020). Jamieson et al. (2018). These authors have proposed an integrated model showing that stress coping strategies modify stress by changing mindsets about the stressful situation. According to this integrated model, numerous studies reveal that, in acute stress conditions, stressful thoughts are associated with negative emotions, but also, by stimulating positive reappraisal of distress and promting a different view of stress, can enhance positive emotions as well (Crum et al., 2014, 2017; Yeager et al., 2016; Keech et al., 2018). During the COVID-19 crisis, a study in Olympic and Paraolympics athletes claims that neuroticism personalities were associated with negative emotions and, in turn, the confinement have a higher impact in their trainng routines and performance (Clemente-Suárez et al., 2020). Furhermore, the study in Olympic and Paraolympics athletes found that there was a strong positive relation between athletes'agreement with the confinement and the perception of institusional support provided.

Additional variables that may mediate the relationship between physical activity or sport and mood states during periods of recovery or stress may be access to psychological support and the athlete's perception of the need to rehabilitate and suspend sports practice in competitive tournaments for a limited time. In a study of athletes facing prolonged recovery from injury, it was shown that the perception of illness has emotional consequences; thus, a high perception of the illness's controllability was associated with lower negative mood (van Wilgen et al., 2010). Regarding access to information or psychological support, Kenttä et al. (2006) found that athletes who obtain continuous feedback about their emotional states during the period of recovery from injury manage to reduce their negative mood states. Mohammed et al. (2018). revealed that during an injury, athletes treated with mindfulness-based techniques reduced their negative moods and were more likely to improve in their injury without suffering from psychological stress.

Although the relationship between physical activity and mood states is widely contrasted, there are divergences about the moderating role of gender. Numerous studies have shown greater stress reaction and negative moods in women than in men in a period of prolonged rest such as that due to injury or absence from competitive events (Di Fronso et al., 2013; Reynoso-Sánchez et al., 2020). Rocheleau et al. (2004) found that women may benefit more than men from improving mood states through physical exercise. Other studies have found no such significant differences between women and men (McAuley and Rudolph, 1995; Reigal et al., 2020). Regarding the impact of utilizing available coping strategies, Reigal et al. (2020) found (in beach handball players) that coping skills are differentially associated with mood states by gender; women gained less stress reduction than men through the use of coping activites. Recently, Clemente-Suárez et al. (2020) demonstrated that, during the COVID-19 pandemic crisis, women athletes scored higher in neuoroticism and psychological inflexibility than men athletes. Therefore, the issue of gender differences in emotions, being still in need of clarification, it might be important to analyse wich factors and mediator variables might promote positive moods in women and which ones reduce their negative moods.

**Figure 1:**
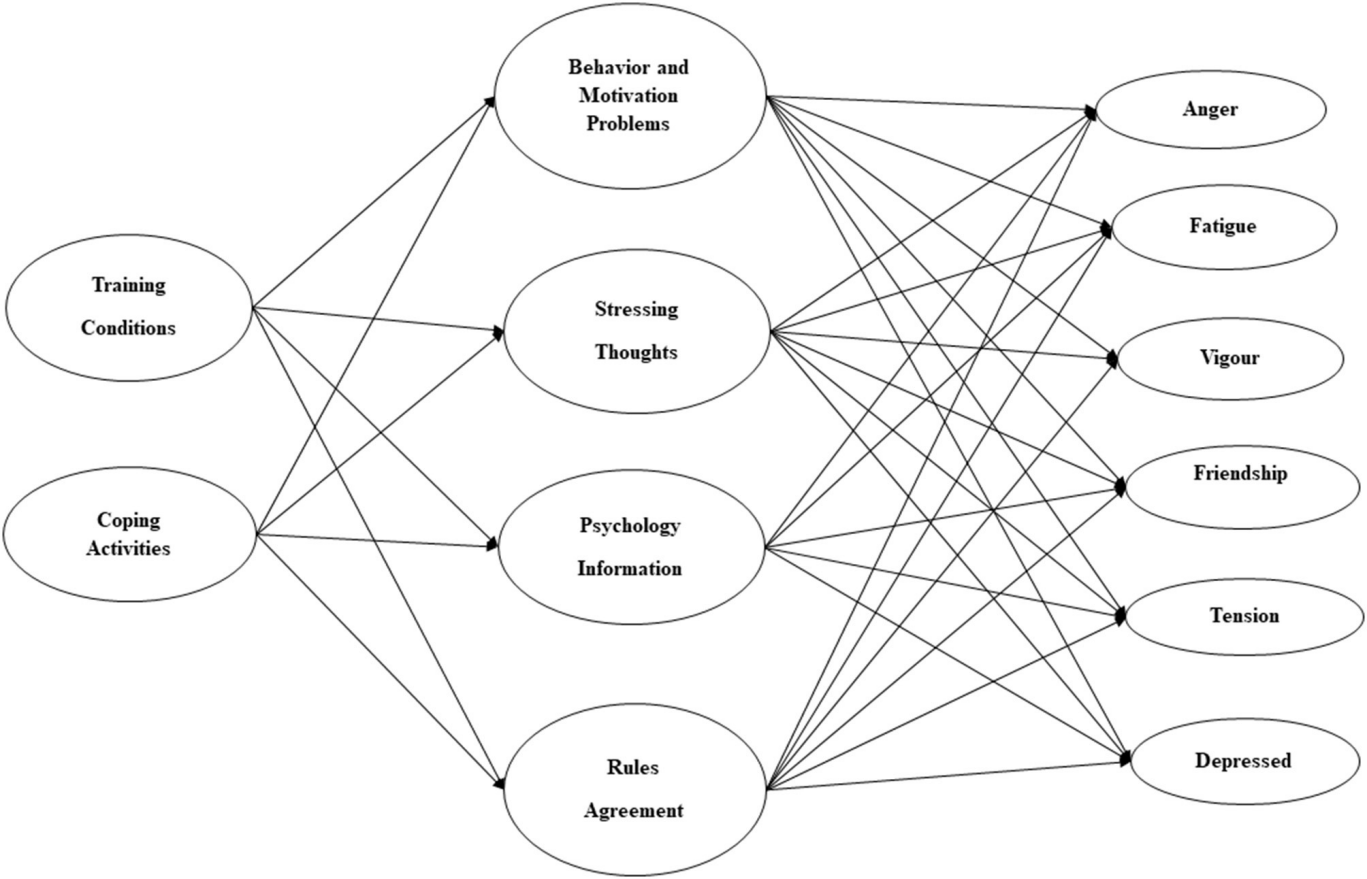
Summary of the hypothesized model.

This study aimed to analyze the effect of the confinement applied in Spain on March 14 2021, to combat the impact of the Covid 19 pandemic, on the behavior thoughts and emotional states of a sample of 1,248 athletes. The main hypothesis was that the maintenance of some training conditions, and the regular performance of coping activities can function as preventive or modulating factors of behavioral and emotional problems, reducing the experience of negative moods in the confinement period. The present study applied a structural equation modeling to examine the relationship between training conditions and coping activities with moods states (anger, fatigue, vigor, feelings of friendship, somatic tension, and depressed moods). The conceptual framework, outlined in Figure 1, assesses the mediating role of behavioral problems, stressful thoughts, seeking psychological support, and attitudes toward confinement rules. Furthermore, by applying multigroup analysis, this study tested possible outcome differences by gender.

## Materials and Methods

### Samples and Procedures

One thousand, two hundred forty-eight athletes participated in the study (Mean age = 22.31 ± 11.49, Female: 53%), who compete at National (*N*: 1,017, Mean age = 21.58 ± 11.42, Female: 52%) and international level (*N*: 231, Mean age = 25.56 ± 11.22, Female: 57%). The sample was drawn from Spanish athletes who compete in individual sports, a detailed description of the athlete's sample grouped by sex, sports and competition level is shown in the Supplementary Table 1. The questionnaires were collected between the 10th of April (2 weeks after the national lockdown) and the 1st of May of 2020.

The study was conducted using a descriptive quantitative methodology based on random, non- purposive sampling, and snow-ball effect. An online *ad hoc* questionnaire was designed (Emotional and Adaptative reactions during confinement COVID-19-RECOVID-19; Jaenes et al., 2020a), to assess competitive Spanish athletes of different sports. In addition to sociodemographic and housing characteristics information, the questionnaire collected information on home training conditions and the performance of coping activities during confinement. These measures of training conditions and coping activities were based on previous studies which examined the relationship between physical exercise, leisure activities, and psychological well-being (Im Yi et al., 2015; García-Castilla et al., 2018). Behavior or motivational problems and stressing thoughts were tested in the questionnaire with some ítems adapted from the Recovery-Stress Questionnaire for Athletes (RESTQ-Sport; Gonzalez-Boto et al., 2008), and adding questions about eating and sleeping pattenrs. Following the recommendations of the Association of the Applied Sport Psychology (Byrd et al., 2020) and the World Health Organization (2020a,c) for the prevention of psychological distress among athletes given the COVID-19 Pandemic, some additional items of the questionnaire were elaborated to evaluate the seeking for psychology advice and the acceptance of confinement rules. The short and validated Spanish version of the Profile of Mood States POMS was used to assess emotional states (Andrade et al., 2010).

The questionnaire included an introduction with the aims of the research, specific instructions to fill it out, and the aims of the survey, ethics information for participants, as well an informed consent for adults and parent permission for athletes under 18. It was necessary to use internet and mainstream media, including WhatsApp^TM^, because of the mobility and personal access limitation imposed during the testing period by the pandemic control efforts. These research methods have been reported as–valid and reliable (Díaz de Rada Igúzquiza et al., 2019).

The questionnaire was initially tested on a sample of 10 athletes (50% were females for each sport) to determine that it was appropriate and understandable: four of which were track and field runners (Mean Age = 27.5, *SD* = 4.79), two were rowers (Mean Age = 27, *SD* = 1.41), two were kayakers (Mean Age = 24.5, *SD* = 0.70) and two were triathletes (Mean Age = 27, *SD* = 12.72). Four coaches and four sport psychologists, blind to the study objectives, were recruited for validation as recommended by Osterlind (1998) with a Likert scale to assess the comprehension and adequacy of the items.

This survey was sent on the Internet using the list of contacts of the Rowing, and Swimming Spanish Federation, Sport and Educations Council of the Andalusian Government, different Andalusian Sport Federations, Andalusian Center of Sport Medicine (CAMD).

### Ethical Statement

The present study was approved by the Ethical Committee of the Andalusian Center of Sport Medicine (CAMD) Project CAMD-PSY2020/01, and was conducted according to the Declaration of Helsinki. The CAMD is a public medical center under the direction of Andalusian Council of Education and Sport, and CAMD's objective is to care for the athlete's physical and psychological health. Participants could withdraw from the survey at any moment without providing any justification, or jeopardizing their status in the organization. To encourage the recruitment of potential future participants, Federations will receive information about results in their particular sport. In addition, Rowing and Swimming Federations will organize a meeting (via Streaming) to inform athletes, coaches and officials. Completing the questionnaire was voluntary, anonymous and confidential. No name was registered. Completion times were between 4 and 8 min. Inclusion criteria were being Spanish and compete at National or International level. Exclusion criteria was to compete at local or regional level.

### Measures

#### Sociodemographic Variables

The survey sought information about the following parameters age, gender, competitive level, and education level. Life and training related information with questions about the house size, the availability of terraces or gardens, and the number of training hours per week are shown in Table 1.

**Table 1:** Descriptive characteristics of the sample by gender.

	**Men**	**Women**	
	***n* (%)**	***n* (%)**	**χ^2^ (*df*)**
Gender	587 (47)	661 (53)	4.38^*^ (1)
Education level			35.487^***^ (3)
Primary school	35^a^ (6)	100^b^ (15.1)	
Secondary school	125^a^ (21.3)	169^a^ (25.6)	
High school	215^a^ (36.6)	189^b^ (28.6)	
University	212^a^ (36.1)	203^b^ (30.7)	
Competition level			1.986 (1)
National	488 (83.1)	529 (80)	
International	99(16.9)	132 (20)	
Garden or outdoor	366 (62.4)	430 (65.1)	0.984 (1)
terrace at home			
Home square meters			1.266 (3)
<70 m^2^	57 (9.7)	60 (9.1)	
Between 70 and 90 m^2^	176 (30)	190 (28.7)	
Between 90 and 120 m^2^	188 (32)	205 (31)	
More than 120 m^2^	166 (28.3)	206 (31.2)	
Weekly training hours			8.607 (5)
<5 h	96 (16.4)	81 (12.3)	
Between 5 and 7 h	167 (28.4)	188 (28.4)	
Between 8 and 10 h	123 (21)	175 (26.5)	
Between 11 and 13 h	106 (18.1)	107 (16.2)	
Between 14 and 16 h	57 (9.7)	62 (9.4)	
More than 16 h	38 (6.5)	48 (7.3)	

*Percentages (%) displayed refer to column percentages*.

* *p-value chi-square < 0.05*;

*** *p-value chi-square < 0.001*.

a and b *show significant difference subset groups*.

#### Training Conditions

Training conditions on confinement were tested by four items, shown in Table 2, availability of equipment at home to train properly, coaches follow up, organization timesheet, and their opinion on their ability to keep their physical fitness. Participants were asked to answer on a four-point Likert scale from 1 (Never) to 4 (Very frequently).

**Table 2:** Item measures mean differences by gender.

	**Men**	**Women**	
	**Mean (*SD*)**	**Mean (*SD*)**	***t*-test (*df*)**
**Training conditions**			
In confinement, you had material to train properly			
	2.37 (0.75)	2.35 (0.75)	0.474 (1,246)
In confinement, you had a follow-up from your coach			
	2.70 (1.05)	2.96 (1.01)	−4.370^***^ (1,246)
In confinement, you have been able to organize yourself to train			
	3.13 (0.80)	3.12 (0.77)	0.194 (1,246)
In confinement, you have maintained physical fitness			
	2.45 (0.92)	2.49 (0.86)	−0.778 (1,246)
**Coping activities**			
Parcel 1: reading, radio, social networks, and cooking			
	2.39 (1.01)	2.69 (0.99)	−5.309^***^ (1,246)
Parcel 2: television, music, mindfulness, videoconferences, languages			
	2.88 (0.67)	3.08 (0.60)	−5.554^***^ (1,246)
Parcel 3: video games, study, yoga, learn something new, draw or paint			
	2.21 (0.99)	2.48 (1.02)	−4.743^***^ (1,246)
**Behavior and motivational problems**			
Do you have manias or rituals that you didn't have?			
	1.42 (0.70)	1.52 (0.81)	−2.303^*^ (1,246)
In general. it has been difficult to stay motivated to train			
	2.64 (1.19)	2.60 (1.21)	0.602 (1,246)
Have you eaten more than you usually eat?			
	1.96 (1.05)	2.09 (1.06)	−2.120^*^ (1,246)
**Stressing thoughts**			
Do you have sleep disturbances/difficulties?			
	2.46 (1.24)	2.60 (1.19)	−2.043^*^ (1,246)
Is it difficult for you to concentrate?			
	2.31 (1.10)	2.47 (1.07)	−2.591^**^ (1,246)
Have you been afraid that a family member would pass away?			
	2.12 (1.10)	2.45 (1.18)	−5.100^***^ (1,246)
**Seek for psychology advice**			
Have you talked to a psychologist during this time?			
	1.08 (0.36)	1.13 (0.47)	−1.964^*^ (1,246)
Have you received any psychological information during this time?			
	1.31 (0.65)	1.41 (0.76)	−2.561^*^ (1,246)
Has the information or psychological advice been useful to you?			
	1.45 (0.88)	1.56 (1.01)	−2.021^*^ (1,246)
**Agreement to confinement rules**			
In confinement, do you think it was necessary for you to have been quarantined?			
	3.22 (1.00)	3.34 (0.94)	−2.188^*^ (1,246)
In confinement, do you have respected the quarantine rules?			
	3.88 (0.38)	3.92 (0.31)	−2.161^*^ (1,246)
In confinement, do you think it was right that the Olympics were suspended?			
	3.68 (0.59)	3.71 (0.56)	−0.900 (1,246)

* *p < 0.05*;

** *p < 0.01*;

*** *p < 0.001*.

#### Coping Activities Utilization

A list of coping strategies was assessed through gathering information about activities such as reading, watching TV, playing video-games, listening to music, practicing relaxation techniques, using social networks, learning something new like cooking, etc. The 15 items coping activities were coded as “1. yes” or “0. no” The 15 items were combined into three parcels shown in Table 2, with five items each to operationalize the latent variable (Ho, 2013). Them the sum of items in each parcel ranked from 0 to 5.

#### Behavior and Motivational Problems

Three items, Table 2, sample the presence of obsessive or compulsive thoughts or behaviors or rituals in confinement, difficulties in maintaining training motivation, and significant variation in food intake from their usual baselines. Participants were asked to answer on a five-point liker scale from 1 (Never) to 5 (Extremely frequently).

#### Stressing Thoughts

Stressing thoughts during the time confined were assessed through three items, Table 2: sleep disturbances, difficulty concentrating, and fear of the death of a family member. Participants were asked to answer on a five-point liker scale from 1 (Never) to 5 (Extremely frequently).

#### Seeking Psychological Information/Support

Psychological information and support to deal with their responses to confinement was evaluated via three items, Table 2, whether the athlete had talked to a psychologist, had received any psychological information, and if they found the psychological advice useful. Participants were asked to answer on a five-point liker scale from 1 (Never) to 5 (Extremely frequently).

#### Acceptance of Confinement Rules

Athletes were asked about their beliefs regarding quarantine, the confinement rules, and if they agreed with the Olympics cancellation (Table 2). Participants were asked to answer on a four-point liker scale from 1 (Never) to 4 (Very frequently).

#### Emotion Scale

A short and validated Spanish version of the Profile of Mood States POMS was used (Andrade et al., 2010). This version has 30 items and six subscales: anger, fatigue, vigor, friendship, tension, and depression, with five answer options from 0 (Not at all) to 5 (Extremely).

### Data Analysis

Statistical analyses were performed using the R language v.3.5.3 (R Development Core Team, 2017) and the RStudio environment v. 1.3.959 (Rstudio Team, 2019), employing a statistical significance at α = 0.05. Descriptive analyses were used to describe the sample characteristics (i.e., sociodemographic). A structural equation model was used to analyze the effect of behavioral, motivation, and stress variables on the emotional states of athletes in confinement, controlling for the effect of age. As multivariate normality distribution of the data, Mardia's coefficient, was statistically significant (*p* < 0.05), the Satorra–Bentler correction of chi-square and standard errors was used (Satorra and Bentler, 2001) and robust versions of CFI, TLI and RMSEA fit indices. The measurement model was tested using a Confirmatory Factor Analysis (CFA), shown in **Table 5**, reaching good levels of fit indices, higher than 0.95 for the comparative fit index (CFI) and Tucker-Lewis index (TLI) and < 0.05 for the square root error of approximation (RMSEA), and the standardized root mean square residual (SMRS) (Hooper et al., 2008), after allowing the correlation between the residuals of some indicators of the scale of emotional states using as criteria the index modification values (Byrne et al., 1989). Previous studies have shown measurement and configural invariance after allowing residuals correlation for the Profile of Mood States (POMS; Terry et al., 2003 Kim and Smith, 2017), and it has a theoretical driven consensus given that some of the subscales measures positive mood states and the others negative mood states (Boyle et al., 2015; Echemendia et al., 2019).

## Results

### Descriptive Statistics and *t*-Tests/χ^2^-Test

Table 1 shows the sociodemographic data for men and women separately. There were differences by gender in age and educational level: the mean age of women was lower (M_women_ = 20.02, *SD* = 9.16) than that of men (M_men_ = 24.88, *SD* = 13.17), and there was a significantly higher percentage of women with a primary education level (15.1%) than men (6%).

Women, see Table 3, performed coping activities more frequently during confinement (*t* = −7.33, *p* < 0.05), used consultation with a psychologist or sought psychological information (*t* = −2.56, *p* < 0.05), and they exhibited higher compliance with confinement rules more (*t* = −2.47, *p* < 0.05). Women suffered greater stress symptoms (*t* = −5.175, *p* < 0.05). On the scale of emotional states, compared to men, women showed significantly higher ratings of negative emotional states.

**Table 3:** Age and scales mean differences by gender.

	**Men**	**Women**	
	**Mean (*SD*)**	**Mean (*SD*)**	***t*-test (*df*)**
Age	24.88 (13.17)	20.02 (9.16)	7.63^***^ (1,246)
Coping activities	7.47 (1.83)	8.25 (1.88)	−7.33^***^ (1,246)
Training conditions	2.66 (0.62)	2.72 (0.58)	−1.946 (1,246)
Behavior and motivational problems	2.00 (0.67)	2.07 (0.70)	−1.573^**^ (1,246)
Stressing thoughts	2.18 (0.73)	2.41 (0.79)	−5.175^***^ (1,246)
Seek for psychology advice	3.83 (1.61)	4.09 (1.92)	−2.56^**^ (1,246)
Agreement to confinement rules	3.59 (0.47)	3.65 (0.44)	−2.47^*^ (1,246)
Emotion Scale			
Anger	1.91 (0.75)	2.13 (0.81)	−4.96^***^ (1,246)
Fatigue	1.90 (0.85)	2.10 (0.89)	−3.98^***^ (1,246)
Vigor	2.49 (0.89)	2.47 (0.85)	0.46 (1,246)
Friendship	2.89 (0.83)	2.97 (0.82)	−1.70 (1,246)
Tension	2.18 (0.98)	2.43 (0.99)	−4.31^***^ (1,246)
Depressed	1.87 (0.87)	2.08 (0.93)	−4.06^***^ (1,246)

* *p < 0.05*;

** *p < 0.01*;

*** *p < 0.001*.

### Correlation Analyses

Table 4 displays the partial correlation, by gender, between the behavioral variables and emotional states, controlling for age, with women on the diagonal and men below the diagonal; and the Cronbach's α for men and women in the diagonal. Across gender, the coping activities were positively associated with anxiety symptoms (*r*_all_ = 0.068, *p* < 0.05), compliance with the rules of confinement (*r*_all_ = 0.78, *p* < 0.01), and with the need to seek psychological support (*r*_all_ = 0.204, *p* < 0.01). Coping activities were positively correlated with both emotional states of vigor (*r*_all_ = 0.134, *p* < 0.05) and friendship (*r*_all_ = 0.171, *p* < 0.05), as well as with emotional states of tension (*r*_all_ = 0.102, *p* < 0.01) and depression (*r*_all_ = 0.060, *p* < 0.05).

**Table 4:** Summary of partial correlation, controlling for age, between measures by gender.

***α _***Women***_*** *** α _***Men***_***	**1**	**2**	**3**	**4**	**5**	**6**	**7**	**8**	**9**	**10**	**11**	**12**
Coping activities	*0.486 0.400*	0.059	−0.003	0.044	0.214^***^	−0.017	0.026	0.048	0.130^**^	0.120^**^	0.109^**^	0.055
Training condition	0.068	*0.651 0.624*	−0.352^***^	−0.266^***^	0.120^**^	0.029	−0.171^**^	−0.199^**^	0.325^**^	0.160^**^	−0.173^**^	−0.225^***^
Behavior and motivation problems	−0.026	−0.427^***^	*0.419 0.390*	0.513^***^	−0.059	−0.027	0.374^***^	0.413^***^	−0.276^***^	−0.073	0.341^***^	0.390^***^
Stressing thoughts	0.049	−0.342^***^	0.506^***^	*0.614 0.485*	0.008	−0.119^**^	0.607^***^	0.564^***^	−0.229^***^	−0.078^*^	0.534^***^	0.567^***^
Seek for psychology advice	0.171^**^	−0.045	0.003	0.079	*0.765 0.732*	0.003	−0.020	−0.012	0.129^**^	0.098^*^	0.049	−0.021
Agreement to rules	0.151^**^	−0.060	−0.112^**^	−0.033	0.031	*0.385 0.379*	−0.149^**^	−0.064	0.024	0.088^*^	−0.044	−0.060
Anger	0.003	−0.260^***^	0.467^***^	0.606^***^	0.078	−0.065	*0.889 0.886*	0.552^***^	−0.137^**^	−0.121^**^	0.547^***^	0.638^***^
Fatigue	0.014	−0.211^***^	0.449^***^	0.481^***^	0.111^**^	−0.045	0.549^***^	*0.897 0.896*	−0.160^**^	0.075	0.550^***^	0.581^***^
Vigor	0.153^**^	0.374^***^	−0.344^***^	−0.258^***^	0.040	0.013	−0.166^**^	−0.213^**^	*0.869 0.876*	0.512^***^	0.043	−0.131^**^
Friendship	0.214^***^	0.196^**^	−0.206^**^	−0.132^**^	0.008	0.073	−0.136^**^	−0.009	0.571^***^	*0.888 0.894*	0.139^**^	0.027
Tension	0.063	−0.111^*^	0.352^***^	0.508^***^	0.039	−0.071	0.587^***^	0.500^***^	0.018	0.102^*^	*0.891 0.900*	0.606^***^
Depressed	0.029	−0.218^**^	0.403^***^	0.499^***^	0.051	−0.037	0.607^**^	0.527^**^	−0.161	−0.061	0.545^***^	*0.821 0.817*
Age _Women_	−0.074	−0.289^***^	0.001	−0.008	0.038	0.048	−0.100^*^	−0.027	−0.084^*^	0.006	−0.088^*^	0.015
Age _Men_	−0.091^*^	−0.247^***^	−0.067	−0.149^**^	−0.052	0.137^**^	−0.184^**^	−0.189^**^	−0.027	0.062	−0.203^**^	−0.124^**^

*Men are down of the diagonal and Women are up of the diagonal. Chronbach's Alpha are displayed on the diagonal (Women are up and Men are down)*.

* *p < 0.05*;

** *p < 0.01*;

*** *p < 0.001*.

Those athletes who had adequate training materials and more monitoring and information from their coaches and federation, reported lower levels of perceived stress (*r*_all_ = −0.387, *p* < 0.01) and had fewer behavioral and motivational problems (*r*_all_ = −0.300, *p* < 0.01). For women and men, the training conditions were positively correlated with the negative moods, and negatively correlated with the positive moods. Whereas, for women and men, motivation and behavior problems, stressful thoughts, and symptoms were negatively correlated with positive emotional states, and positively correlated with experiencing negative moods. The need to seek psychological support was positively associated, in both women and men, with emotional states of friendship (*r*_all_ = 0.062, *p* < 0.05), and vigor (*r*_all_ = 0.086, *p* < 0.01). Agreement with the rules of confinement was negatively correlated with the perception of behavioral and motivational problems (*r*_all_ = −0.065, *p* < 0.05), stress symptoms (*r*_all_ = −0.068, *p* < 0.05), and with the emotional state of anger (*r*_all_ = −0.099, *p* < 0.01), and positively correlated with the emotional state of friendship (*r*_all_ = 0.086, *p* < 0.01).

### Structural Equation Model Analyses

The structural equation model, see Table 5, reached an acceptable model fit, exceeding 0.9 for CFI, TFI, and <0.05 for RMSE and SMRS <0.06 (Kline, 2013).

**Table 5:** Summary of model fit indices for CFA and Structural nested models.

**Models**	**S-Bχ^2^**	***df***	**R-CFI**	**R-TLI**	**R-RMSEA**	**SRMR**	**ΔCFI**
CFA	1,906.838^***^	906	0.956	0.950	0.032	0.042	
SM	2,380.032^***^	960	0.940	0.932	0.037	0.055	−0.016
MSM	3,371.723^***^	1,920	0.938	0.930	0.037	0.057	−0.002
MSMER	3,574.363^***^	1,964	0.932	0.925	0.038	0.058	−0.006

*** *(p-value chi-square < 0.001); CF, Configural model; SM, Structural model; MSM, Multigroup Structural model; ST, Strict model; ERMSM, Equality of regression path and intercepts in MSM; S-Bχ^2^, Satorra-Bentler chi-square; df, degrees of freedom; R-CFI, Robust CFI; R-TLI, Robust TLI; R-RMSEA, Robust RMSEA*.

The age of the athletes, shown in Table 6, was negatively associated with behavioral or motivational problems (β = −0.209, *p* < 0.01) and levels of perceived anxiety in confinement (β = −0.255, *p* < 0.01). In contrast, the age of the athletes was positively related to the respect of the confinement rules (β = 0.147, *p* < 0.01). Also, the negative emotional states decreased significantly with the age of the athletes.

**Table 6:** Summary of path coefficients from age among male and female.

	**All β (S.E.)**	**Women β (S.E.)**	**Men β (S.E.)**	**Z-score**
**Age to**				
Behavior and motivational problems	−0.209^***^ (0.029)	−0.171^***^ (0.042)	−0.240^***^ (0.041)	−1.158
Stressing thoughts	−0.255^***^ (0.025)	−0.132^***^ (0.035)	−0.322^***^ (0.034)	−3.926^***^
Seek for psychology advice	0.016 (0.025)	0.085^*^ (0.040)	−0.034 (0.035)	−2.259^*^
Agreement to confinement rules	0.147^***^ (0.037)	0.086 (0.055)	0.241^***^ (0.052)	2.045^*^
Anger	−0.080^**^ (0.025)	−0.086^**^ (0.031)	−0.050 (0.041)	0.687
Fatigue	−0.064^*^ (0.026)	−0.043 (0.036)	−0.065 (0.040)	−0.414
Vigor	−0.075^*^ (0.034)	−0.086 (0.044)	−0.108^*^ (0.052)	−0.321
Friendship	−0.006 (0.033)	−0.012 (0.045)	−0.021 (0.052)	−0.132
Tension	−0.098^***^ (0.026)	−0.091^**^ (0.034)	−0.094^*^ (0.043)	−0.042
Depression	−0.003 (0.028)	0.016 (0.035)	0.002 (0.046)	−0.246

*Z-score diplayed differences by gender (Men vs. Women)*.

* *p < 0.05*;

** *p < 0.01*;

*** *p < 0.001*.

Controlling for age, shown in Table 7, adequate training conditions and follow-up by a coach were associated with a reduction in lack of motivation or the presence of behavior problems (β = −0.877, *p* < 0.01) and anxiety levels (β = −0.634, *p* < 0.01). Coping activities were associated with higher stress symptoms (β = 0.179, *p* < 0.01) and the need to search for information or psychological support (β = 0.336, *p* < 0.01). Those athletes who performed a higher number of coping activities showed greater compliance with confinement rules (β = 0.165, *p* < 0.01).

**Table 7:** Summary of path coefficients from coping and training by gender.

	**All β (S.E.)**	**Women β (S.E.)**	**Men β (S.E.)**	**Z-score**
**Coping activities to**				
Behavior and motivational problems	0.053 (0.044)	0.077 (0.065)	0.011 (0.059)	−0.757
Stressing thoughts	0.179^***^ (0.044)	0.231^***^ (0.063)	0.069 (0.061)	−1.857
Seek for psychology advice	0.336^***^ (0.044)	0.327^***^ (0.056)	0.302^***^ (0.074)	−0.264
Agreement to confinement rules	0.165^**^ (0.060)	−0.048 (0.084)	0.426^***^ (0.082)	4.024^***^
**Training conditions to**				
Behavior and motivational problems	−0.877^***^ (0.032)	−0.897^***^ (0.045)	−0.843^***^ (0.044)	0.861
Stressing thoughts	−0.634^***^ (0.033)	−0.641^***^ (0.047)	−0.595^***^ (0.044)	0.712
Seek for psychology advice	0.043 (0.0335)	0.093^*^ (0.046)	0.009 (0.054)	−1.194
Agreement to confinement rules	0.012 (0.051)	0.085 (0.068)	−0.104 (0.074)	−1.876

* *p < 0.05*;

** *p < 0.01*;

*** *p < 0.001*.

Controlling for age, the stress symptoms of the athletes, shown in Table 8, were associated with a greater perception of negative emotional states. The highest frequency of behavior and motivation problems, was positively associated with the negative emotional states of anger (β = 0.159, *p* < 0.01), fatigue (β = 0.450, *p* < 0.01), and depression (β = 0.455, *p* < 0.01); and, in turn, motivation or behavior problems significantly reduced positive emotional states of vigor (β = −0.484, *p* < 0.01) and friendship (β = −0.178, *p* < 0.01). Those athletes who sought more information or psychological support reported higher levels of positive vigor emotions (β = 0.075, *p* < 0.01) and friendship (β = 0.068, *p* < 0.05). Agreement with the rules of confinement was positively related to the perception of emotional states of friendship (β = 0.145, *p* < 0.01) and negatively associated with the emotional states of anger (β = −0.116, *p* < 0.01).

**Table 8:** Summary of direct path coefficients to mood states by gender.

	**All β (S.E.)**	**Women β (S.E.)**	**Men β (S.E.)**	**Z-score**
**Behavior and motivational problems to**				
Anger	0.159^***^ (0.042)	0.087 (0.059)	0.256^***^ (0.058)	2.051^*^
Fatigue	0.276^***^ (0.045)	0.246^***^ (0.060)	0.325^***^ (0.062)	0.914
Vigor	−0.484^***^ (0.046)	−0.485^***^ (0.065)	−0.449^***^ (0.062)	0.397
Friendship	−0.178^***^ (0.047)	−0.153^*^ (0.068)	−0.182^**^ (0.060)	−0.317
Tension	0.046 (0.042)	0.061 (0.058)	0.065 (0.059)	0.038
Depression	0.236^***^ (0.044)	0.231^***^ (0.062)	0.241^***^ (0.058)	0.116
**Stressing thoughts to**				
Anger	0.508^***^ (0.041)	0.535^***^ (0.053)	0.461^***^ (0.062)	−0.911
Fatigue	0.450^***^ (0.044)	0.504^***^ (0.055)	0.369^***^ (0.065)	−1.580
Vigor	−0.068 (0.048)	−0.008 (0.066)	−0.174^**^ (0.064)	−1.808
Friendship	−0.021 (0.047)	0.032 (0.065)	−0.112 (0.064)	−1.580
Tension	0.489^***^ (0.043)	0.519^***^ (0.054)	0.422^***^ (0.064)	−1.163
Depression	0.455^***^ (0.044)	0.486^***^ (0.058)	0.410^***^ (0.064)	−0.875
**Seek for psychology advice to**				
Anger	0.046 (0.028)	−0.011 (0.041)	0.108^**^ (0.042)	2.051^*^
Fatigue	0.046 (0.026)	−0.031 (0.035)	0.139^***^ (0.041)	3.154^**^
Vigor	0.068^*^ (0.031)	0.107^*^ (0.041)	0.027 (0.038)	−1.414
Friendship	0.075^**^ (0.029)	0.107^*^ (0.043)	0.000 (0.042)	−1.787
Tension	0.068^*^ (0.031)	0.060 (0.043)	0.043 (0.040)	−0.281
Depression	0.053 (0.030)	−0.003 (0.044)	0.068 (0.042)	1.154
**Agreement to confinement rules**				
Anger	−0.116^**^ (0.042)	−0.184^**^ (0.062)	−0.091 (0.057)	1.107
Fatigue	−0.060 (0.041)	−0.078 (0.053)	−0.073 (0.065)	0.058
Vigor	0.014 (0.041)	0.008 (0.059)	0.071 (0.056)	0.773
Friendship	0.145^***^ (0.042)	0.130^*^ (0.057)	0.197^**^ (0.064)	0.775
Tension	−0.045 (0.042)	−0.025 (0.060)	−0.062 (0.063)	−0.424
Depression	−0.069 (0.042)	−0.077 (0.059)	−0.092 (0.062)	−0.176

* *p < 0.05*;

** *p < 0.01*;

*** *p < 0.001*.

The total indirect effects, shown in Table 9, of the training conditions and the frequency of use of coping activities on the subject's emotional states, were analyzed using a mediation model. Adequate training and monitoring conditions by the trainer were associated with a lower reported experience of negative emotional states. Men and women reported that the training conditions and the monitoring of their coaches were positively associated with the positive emotional states of vigor and friendship. However, the frequency of use of coping activities was associated with higher negative emotional states.

**Table 9:** Summary of indirect path coefficients to mood states by gender.

	**All β (S.E.)**	**Women β (S.E.)**	**Men β (S.E.)**	**Wald test**
**Coping activities to**				
Anger	0.096^***^ (0.029)	0.136^***^ (0.041)	0.028 (0.044)	2.968
Fatigue	0.101^***^ (0.029)	0.129^**^ (0.043)	0.040 (0.045)	2.209
Vigor	−0.010 (0.024)	−0.005 (0.034)	0.022 (0.037)	0.279
Friendship	0.034 (0.018)	0.025 (0.024)	0.074^*^ (0.035)	1.405
Tension	0.100^***^ (0.026)	0.146^***^ (0.040)	0.016 (0.037)	5.701^*^
Depression	0.093^**^ (0.029)	0.133^**^ (0.043)	0.012 (0.043)	4.103^*^
**Training conditions to**				
Anger	−0.462^***^ (0.027)	−0.438^***^ (0.038)	−0.480^***^ (0.037)	0.665
Fatigue	−0.526^***^ (0.027)	−0.553^***^ (0.038)	−0.485^***^ (0.037)	1.634
Vigor	0.472^***^ (0.030)	0.450^***^ (0.042)	0.475^***^ (0.042)	0.267
Friendship	0.174^***^ (0.031)	0.138^**^ (0.045)	0.200^***^ (0.041)	1.115
Tension	−0.349^***^ (0.029)	−0.385^***^ (0.039)	−0.299^***^ (0.043)	2.439
Depression	−0.495^***^ (0.029)	−0.526^***^ (0.041)	−0.437^***^ (0.040)	2.262

* *p < 0.05*;

** *p < 0.01*;

*** *p < 0.001*.

Two nested models were used to compare the path coefficients between men and women, differences in the χ^2^ (Δχ^2^) and the degree of freedom (Δ*df*) were used to compare the models with the goodness of fit to determine the model that best fit the data (Satorra, 2000; Byrne and Stewart, 2006). The multigroup model not constrained by gender represented a significant change concerning the constrained model assuming equal coefficients regression path and intercepts between the gender groups (Δχ^2^ = 202.64, Δ*df* = 44, *p* < 0.01). To check the differences of the structural path coefficients between genders, we used the z-score test for the critical ratio differences (Clogg et al., 1995; Paternoster et al., 1998; Yan et al., 2013), the differences between coefficients were reported as significant for *p* < 0.05, using two-tailed hypothesis (Hair et al., 2010).

There were differences by gender on the path of age to stressing thoughts (z = −3.926, *p* < 0.001), seek for psychology advice (z = −2.259, *p* < 0.05), and agreement to confinement rules (z = 2.045, *p* < 0.05). With age, women were more likely to seek psychological advice than men; whereas, with age, men more likely to agree with confinement rules and less likely to experience stress than women. Controlling for age, the results showed that the structural path from coping to rules compliance was significantly different by gender (z = 4.024, *p* < 0.01). The coping activities were positively associated with respect and agreement with the rules of confinement among men (β = 0.426, *p* < 0.01) but not among women (β = −0.048, *p* = 0.56). The effect of the behavior problems on anger was significantly different by gender (z = 2,051, *p* < 0.05); the men who reported having behavioral and motivational problems presented greater feelings of anger (β = 0.256, *p* < 0.01), while this association was not observed among women (β = 0.087, *p* = 0.14). Also, there were differences by gender in the association between the search for psychological information with the reported states of anger (z = 2,051, *p* < 0.05). Men who sought more psychological support were likely to report a greater feeling of anger (β = 0.108, *p* < 0.01), whereas, this relationship was not observed in women (β = −0.011, *p* = 0.78). The seek for psychological support was associated with the state of fatigue significantly different by gender (z = 3.154, *p* < 0.01); seeking psychological support was related to higher levels of fatigue in men (β = 0.139, *p* < 0.01), but it was not associated with fatigue in women (β = −0.043, *p* = 0.23).

To compare the indirect effects by groups, we performed a multiple mediator model with multiple groups by gender, using the Wald test for testing the group difference in the indirect path effects (Ryu and Cheong, 2017; Klopp, 2019). In the group of women, the frequency of coping activities was significantly associated with the increase in negative emotional states among women; however, in men, no significant relationship was observed between coping activities and emotional states. The frequency of coping activities was positively related to the emotion of friendship among men (β = 0.074, *p* < 0.01), while they were not associated in women (β = 0.026, *p* = 0.60). The indirect effect of the frequency of coping activities was significantly different between men and women on the emotional states of tension (Wald = 5.701, *p* < 0.05) and depression (Wald = 4.103, *p* < 0.05). The indirect effect of training conditions on negative emotional states were not significantly different between genders; that is, equally among women and men, the better the training conditions, the lower the experience of negative moods. Likewise, there were no gender differences in the indirect effect of the training conditions on the level of friendship and vigor. The training conditions were positively associated, equally for women and men, with the positive moods.

## Discussion

Due to the severe health impact of the COVID-19 pandemic in ealy 2020, the Spanish government-imposed (via State of Alarm Declaration) a radical confinement of the Spanish population at their homes from March 14, 2020, until June 21, 2020. The emotional impact of the associated mobility restrictions and social isolation measures on athletes was especially significant. Along with the difficulty of living in isolation at home, athletes had to radically break with their usual training routines (Chirico et al., 2020) and thus possibly threaten the viability of such effort demanding careers.

### Emotional Risk and Protective Factors

The main purpose of this study was to obtain trustworthy, real-time information about the risk and protective factors that can influence emotional symptoms and disorders potentially caused by the confinement of these Spanish athletes who compete nationally and internationally. We collected information about the following parameters:

#### Socio-Demographic and Living Variables

We evaluated the socio-demographic characteristics and the actual living conditions of the athletes during confinement. By gender, we found that women athletes had a lower level of education than men, a difference that was partially explained by the fact that the women were younger on average and so they were more likely to be in primary and secondary education than men. According to Fuentes-García et al. (2020) in a sample of chess players during the COVID-19 confinement, those who had a higher academic level of education scored lower in level of anxiety. Probably the age of the participants could help explain some differences between men and women in the educational level and the appearance of emotional disorders as a consequence of the lockdown (Brooks et al., 2020). As the results of the correlation analyses showed a significant association between age and the behavioral and emotional measures, all the further analysis were controlled by age.

#### Training Conditions

We found no significant difference between male and female athletes, in terms of weekly training frequency, or in the basic space (in square meters) available to them in their confinement residence, or in whether or not they had additional space in the form outdoor patio or terrace. During the confinement at home, men and women had similar conditions of space and training routine. In our sample, neither having or not having a terrace nor access and use of training hours did not seem to explain the differences found in the athletes mood states. Recent studies show that having even minimal physical activity was positively associated with the quality of life during the confinement by COVID-19 (Slimani et al., 2020). Such findings supported the many recommendations made to engage in physical activity during t COVID-19 situation with home-based exercises being always included (Chtourou et al., 2020; Hammami et al., 2020).

#### Coach-Athlete Interactions

Our results show that Spanish coaches maintained a positive ongoing follow-up with their trainees, and monitored equally the training of men and women. This coach follow-up, along with the provision of a necessary minimum of sports equipment, helped athletes to feel less stress, avoid behavioral and motivational problems, and reduce negative emotional states and increase their positive states in both, men and women. It had already been demonstrated that providing clear and accessible information, clear action guidelines and ongoing transparent information about the COVID-19 situation, reduced stressful reactions on the general population (Brooks et al., 2020).

#### Stress Risk Factors

Brooks et al. (2020), in a systematic review of the psychological impact of quarantine in non-athletes, found some critical stressors: inadequate supplies, and insufficient information, among others, made difficult to cope with the confinement. Similarly, our findings show that suitable training materials, coach follow up and the provision of information, were protective factors against behavioral, motivational, and stress problems. German studies show that at the beginning of the COVID-19 pandemic, maintaining a healthy lifestyle might have a preventive effect on the development of anxiety disorders (Petzold et al., 2020). A recent meta-analysis found that, regardless of age and sex, people with higher exercise levels. Had a reduced risk of depression (Stubbs et al., 2016). Our study shows that, as hypothesized, training conditions had a great capacity to modulate negative mood states and increase positivity in competitive athletes (Jaenes et al., 2020b).

#### Coping Activities: Gender Differences

There were differences by gender in the number and type of the coping activities that athletes engaged in during the confinement. Women made greater use of coping activities, such as using social networks, accessing the internet, watching television, or listening to music, among others. However, the multigroup SEM analysis shows that, in women, the use of coping activities was associated with increased anxiety, stress, fatigue, tension and depression. It might be that, at least for women, the increase in the use of coping activities was an attempt to reduce their elevated negative moods (Shechter et al., 2020) found that, during the COVID-19 pandemic, healthcare workers who screened positive for stress and depressive symptoms reported engaging in more coping behaviors.

#### Psychological Counseling

Results about seeking psychological advice show that those who looked for psychological information, and tried to consult with psychologists, increased their state of vigor and decreased anger. As different authors recommend (Reardon et al., 2020; Schinke et al., 2020), it has been shown that including in the disaster situation's help package a First Psychological Aid simple protocol which helps affected individuals by (1) looking at practical ways to support people suffering from serious distress reactions, (2) listening to needs and concerns and (3) linking people with social support, information and professional help is very effective in populations under stress (Mehrsafar et al., 2020).

#### Response to the Cancellation of the 2020 Tokyo Games

In our study, both women and men reported a highly agreement with the cancelation of the 2020 Games due to the coronavirus crisis. Clemente-Suárez et al. (2020) also found a high agreement on the suspension of the Games in a sample of Spanish Olympic and Paralympic athletes. Bear in mind that the rules of forced confinement to private homes for the Spanish population severely limited the practice of outdoor activities, and training in sports centers. In our study, women showed greater respect and acceptance the rules of confinement than men, in line with what was found in Spanish population by De La Vega et al. (2020). Our study shows that the agreement with, and acceptance of the rules of confinement decreased symptoms of stress and anger, and increased feelings of friendship. Besides, respect for the rules increased with age; which also acts a modulator of states of fatigue and tension.

### Conclusions

In conclusion, the condition of confinement during the COVID-19 had a high emotional impact on Spanish athletes. Women suffered more significant anxiety, stress, fatigue, tension and depression than men; but there were no significant differences in terms of the positive states such as friendship and vigor. Similar findings have been reported in a sample of Spanish Olympic and Paralympic athletes facing the 2021 Tokyo Olympiad in the COVID-19 pandemic (Clemente-Suárez et al., 2020), women scored higher in negative emotions (neuroticism and inflexibility) than men. These results fit those found in a sample of Spanish competitive swimmer, where women also scored higher on negative mood states such as anger, fatigue, tension and depression (Jaenes et al., 2020a). Again, no significant differences were found in terms of positive mood states. As in our current study, the negative mood states were associated with a lack of motivation to train, the presence of some behavioral disorders, and experiencing stressful thoughts (Jaenes et al., 2020a).

### Lessons for the Future

As the initial global impact of the COVID-19 pandemic, gets to be widely known sports organizations and authorities at all levels of competition should be encouraged to develop guidelines to confront adverse mass situations like the COVID pandemic. This study provides evidence that suggests some measures that may reduce the psychological and emotional burden of athletes. Initiatives such as: (1) providing adequate equipment for indoor training, (2) giving clear guidance about potential actions, (3) supporting the value of public health measures such as confinements, (4) and facilitating access to psychological information and consultations (Reardon et al., 2020) have demonstrated efficacy andeffectiveness in a robust number of studies.

This study also shows that coaches follow-up decreases behavioral and motivational problems. Such connection between coaches and athletes during confinement periods, should provide realistically and trustworthy information about training, health, and psychological support.Transparent, and clear communication from clubs and/or federations helps athletes to maintain psychological stability, it increases their motivation, decreases negative emotions and sensations, and enhances vigor as a positive factor. Our study also shows that the use of coping strategies helped athletes accept the rules of confinement, the loss of mobility and freedom, and the restrictions caused by the atypical situation. In turn, the acceptance confinement rules helps to improve positive emotional states and reduces anger symptoms. Thus, psychologists, coache, and sports authorities would be vital in providing media and social media with rapid access to valid information about confinement rules and coping resources. Information about the proper use of social media, and of easily available coping activities to mitigate negative moods should be essential. Moreover, equally important is an ongoing and transparent discussion of both the negative and positive consequences of WhatsApp, Twitter, Instagram, and other popular networks among athletes and young people. Throughout social media, coaches, and trainers could be creative, in challenging and encouraging athletes to compete among them in different skills-based games, designing positive environments within the athletes' communities.

The gender specific findings of our study, support additional gendered actions by sports authorities. Women's higher scores in negative emotional states, and willingness to seek and accept psychological support could encourage clubs, federation or sport authorities in general to provide psychological information and guidelines and easy access to psychological consultation to manage their negative emotions. Blogs or online-chat channels might be used to provide peer support and to share additional information uploaded by the sports federations and health authorities.

There are some lessons from our study that could provide a framework to deal with the emotional consequences for large populations, of the additional crises that globalization, climate change, other pandemics, mass migrations environmental catastrophes etc. will undoubtedly bring about. Our study helped us move from the common idea of expecting some form of post-traumatic stress disorder to an actual description of a matrix of risk and protective factors that could be addressed in real-time with high yields for relatively moderate investments. A strategy worth replicating in the many crises of contemporary life emerges from our findings that should be replicated. In the many mass adverse events that contemporary life brings about: (1) Do real-time early assessment and repeat them so you can modify your intervention package from real data. (2) Integrate the response of the different level involved authorities so that to the maximum degree possible information is frequent, consistent, clear, and communicated by people close to the population at risk. In our study, the value of the coach's follow-up may have been determined by their familiarity with their subjects, and the credibility of their information, as much as for their attention to equipment and training needs. (3) Provide some training equipment, this simple resource had a huge impact in maintaining the morale of athletes very frightened about their professional futures. (4) Promote easy, familiar conventional community strategies and resources as coping strategies; they might not prevent stress but help people (in our case) to cope with higher levels of emotional upheaval. Reading, TV, music, learning new things, relaxation techniques which can be easily disseminated through social media. (5) Use of professional resources (psychological consultation for example) should be provided, but maybe not as a standard prescription for all. In our study, women sought and made better use of such resource than men. It is worth digging into this finding in search of ways to make it easy for all to access it if and when needed. There is no question in our minds that better methods of emotional protection of people in crises will be one of the enduring silver linings of this otherwise devastating pandemic.

### Limitations

The main limitation of this study is the lack of follow-up of the initial survey findings over the confinement and post-confinement periods. The cross-sectional design used in this study has potential bias such as differences in the characteristics of responders and non-responders. Hence, the results of this study should not be interpreted in causal terms, but this cross-sectional analytical study can provide information on the association between risk factors and psychological health outcomes. Future research would require a longitudinal study to analyze changes in emotional states throughout the confinement period and—more importantly- its connection with athletic performance. Adequate experimental designs would allow to assess the impact of the above-mentioned measures on athletic performance and contrast the relative effect of each intervention or group of interventions. Future research should compare the psychological and emotional effects of confinement on individual and team athletes, as well as the differences between different sports specialties, in search of an evidence-based set of intervention for each sport specialty.

## Data Availability Statement

The raw data supporting the conclusions of this article will be made available by the authors, without undue reservation.

## Ethics Statement

The studies involving human participants were reviewed and approved by Centro Andaluz de Medicina del Deporte. Written informed consent to participate in this study was provided by the participants' legal guardian/next of kin.

## Author Contributions

JJ conceived the study, designed the questionnaire, collected the data, and wrote the initial manuscript. DA analyzed the data, wrote the results and discussion, and provided critical revisions on the successive drafts. MT provided a final critical edit, reorganized the linking and definition of variables, analyses and findings, and additional meanings of findings added recommendations and did a complete English revision. RG, AC, FG, and FL analyzed initially the data and review the proposed model. AM provided critical revisions on the final drafts. All authors read and approved the final manuscript.

## Conflict of Interest

The authors declare that the research was conducted in the absence of any commercial or financial relationships that could be construed as a potential conflict of interest.
