# Supplementary material for: Emotional Reactions and Adaptation to COVID-19 Lockdown (or Confinement) by Spanish Competitive Athletes: Some Lesson for the Future
Source: Front Psychol. 2021 May 26;12:621606. doi: 10.3389/fpsyg.2021.621606 (PMC8187575; doi:10.3389/fpsyg.2021.621606)
Supplement: Supplementary file 1 [file Table_1.docx]

Supplementary Table I. Frequency and percentages by category, sports and gender.

|  | **National** | | **International** | |  |
| --- | --- | --- | --- | --- | --- |
| **Sports** | **Men** | **Women** | **Men** | **Women** | **Total** |
| Swimming | 266 (37%) | 347 (48%) | 39 (5%) | 67 (9%) | 719 (57%) |
| Rowing | 124 (53%) | 70 (30%) | 21 (9%) | 19 (8%) | 234 (18%) |
| Athletism | 30 (32%) | 33 (35%) | 14 (15%) | 17 (18%) | 94 (7%) |
| Triathlon | 21 (55% | 11 (29%) | 4 (11%) | 2 (5%) | 38 (3%) |
| Gymnastics | 2 (6%) | 27 (84%) | 0 | 3 (9%) | 32 (2%) |
| Canoeing | 11 (25%) | 16 (36%) | 8 (18%) | 9 (20%) | 44 (3%) |
| Racquet sports | 13 (43%) | 7 (23%) | 5 (17%) | 5 (17%) | 30 (2%) |
| Outdoor sports | 7 (28%) | 15 (60%) | 0 | 3 (12%) | 25 (2%) |
| Combat sports | 4 (24%) | 2 (12%) | 4 (24%) | 7 (41%) | 17 (1%) |
| Open seas sports | 10 (67%) | 1 (7%) | 4 (27%) | 0 | 15 (1%) |
| **Total** | 488 (39%) | 529 (42%) | 99 (8%) | 132 (11%) | 1248 |

***Note:*** *Row percentages are in brackets.*
